# Gene Expression Studies in Formalin-Fixed Paraffin-Embedded Samples of Cutaneous Cancer: The Need for Reference Genes

**DOI:** 10.3390/cimb43030151

**Published:** 2021-11-30

**Authors:** Omar García-Pérez, Leticia Melgar-Vilaplana, Elizabeth Córdoba-Lanús, Ricardo Fernández-de-Misa

**Affiliations:** 1 Research Unit, Hospital Universitario Nuestra Señora de Candelaria, Ctra. Gral. del Rosario, 145, 38010 Santa Cruz de Tenerife, Spain; omargp6@gmail.com; 2 Universidad de La Laguna, Calle Padre Herrera, s/n, 38200 San Cristóbal de La Laguna, Spain; 3 Instituto Universitario de Enfermedades Tropicales y Salud Pública de Canarias (IUETSPC), 38296 San Cristóbal de La Laguna, Spain; 4 Pathology Department, Hospital Universitario Nuestra Señora de Candelaria, Ctra. Gral. del Rosario, 145, 38010 Santa Cruz de Tenerife, Spain; leticiamvfreedom@hotmail.com; 5 Dermatology Department, Hospital Universitario Nuestra Señora de Candelaria, Ctra. Gral. del Rosario, 145, 38010 Santa Cruz de Tenerife, Spain

**Keywords:** reference gene, malignant melanoma, cutaneous squamous cell carcinoma, qPCR, formalin-fixed paraffin-embedded

## Abstract

Formalin-fixed paraffin-embedded (FFPE) tumour samples may provide crucial data regarding biomarkers for neoplasm progression. Analysis of gene expression is frequently used for this purpose. Therefore, mRNA expression needs to be normalized through comparison to reference genes. In this study, we establish which of the usually reported reference genes is the most reliable one in cutaneous malignant melanoma (MM) and cutaneous squamous cell carcinoma (CSCC). *ACTB*, *TFRC*, *HPRT1* and *TBP* expression was quantified in 123 FFPE samples (74 MM and 49 CSCC biopsies) using qPCR. Expression stability was analysed by NormFinder and Bestkeeper softwares, and the direct comparison method between means and SD. The in-silico analysis with BestKeeper indicated that *HPRT1* was more stable than *ACTB* and *TFRC* in MM (1.85 vs. 2.15) and CSCC tissues (2.09 vs. 2.33). The best option to NormFinder was *ACTB* gene (0.56) in MM and *TFRC* (0.26) in CSCC. The direct comparison method showed lower SD means of *ACTB* expression in MM (1.17) and *TFRC* expression in CSCC samples (1.00). When analysing the combination of two reference genes for improving stability, NormFinder indicated *HPRT1* and *ACTB* to be the best for MM samples, and *HPRT1* and *TFRC* genes for CSCC. In conclusion, *HPRT1* and *ACTB* genes in combination are the most appropriate choice for normalization in gene expression studies in MM FFPE tissue, while the combination of *HPRT1* and *TFRC* genes are the best option in analysing CSCC FFPE samples. These may be used consistently in forthcoming studies on gene expression in both tumours.

## 1. Introduction

The incidence and mortality rate in cutaneous malignant melanoma (MM) and cutaneous squamous cell carcinoma (CSCC) continue increasing every year. [1,2]. Progress has been made in the search of biomarkers as potential predictors for progression to metastatic disease, resistance to treatment and relapse of the disease [3]. Nevertheless, behaviour of the disease in each patient remains largely unpredictable. Thus, further identification of multiple co-expressed biomarkers or characteristic biomarker’s patterns [3,4,5] are crucial for an accurate approach to patients suffering cancer.

Formalin fixation and paraffin-embedding (FFPE) for biopsies or excisions is the main tissue preservation technique [6,7] and a useful tool for retrospective studies regarding skin cancer. It allows tissue samples to be kept for long periods of time, but a significant degradation of nucleic acids along the procedure is the main handicap [8]. It may be due to cross-linking and/or oxidative deamination of mRNA. This issue may account for poor mRNA quality and integrity that hampers subsequent gene expression studies. An improved and robust technique allowing FFPE tissue to be reliably analysed for its RNA content will facilitate the design of useful retrospective studies.

The real time PCR is the most accurate and reliable molecular biology technique in gene expression studies for validation of candidate biomarkers [9,10]. However, in these kinds of studies, it is important to consider several subjects: the quality of the sample, the RNA isolation procedure, the stability and degradation of the RNA, the retrotranscription to cDNA, and the qPCR technique [11]. Thus, comparing the relative mRNA expression of a series of samples needs to normalize the quantitative variations of isolated RNA, the integrity of the RNA and the efficiency of the reverse transcription [12] from one sample to another. The use of reference genes as internal controls is the most common method for normalizing mRNA data but its use needs to be experimentally validated for specific tissues or cell types and specific experimental designs [13]. Unfortunately, these issues are still largely ignored, and many reported studies contain poorly normalized qPCR data [14]. The expression of reference genes should remain constant among different tissues and under different experimental conditions [15]. Otherwise, gene expression studies report on misleading conclusions [16,17,18]. The most widely reported reference genes are housekeeping genes such as glyceraldehyde 3-phosphate dehydrogenase (*GAPDH*), β-actin (*ACTB*) and 18S rRNA ribosomal RNA (*18S* rRNA), but they still lack conclusive validation of its robustness [19].

Definite results concerning reference genes suitable for gene expression studies in FFPE samples of CSCC and MM are scarce. *ACTB*, hypoxanthine phosphoribosyltransferase 1 (*HPRT1*), TATA-box binding protein (*TBP*) and transferrin receptor (*TFRC*) genes were reported to show a minimal average variation in FFPE and frozen tissue samples of melanoma and other diseases [20,21,22,23]. *HPRT1* stably expresses in melanocytic cell lines [24] as well as in laryngeal cancer [25]; as does the *ACTB* gene in FFPE samples of lung squamous cell carcinoma [26,27], and *TBP* and *TFRC* in FFPE breast cancer samples [21,28]. The aim of the current study is to evaluate the usefulness of *ACTB*, *HPRT1*, *TBP* and *TFRC* as candidate reference genes for mRNA expression studies, including CSCC and MM FFPE samples. Establishing the best reference gene will improve the quality of forthcoming expression studies on CSCC or MM samples.

## 2. Materials and Methods

### 2.1. Patients and Study Samples

One hundred and twenty-three FFPE skin samples corresponding to 123 patients were included (49 CSCC and 74 MM). Patients were followed by the Dermatology Department of Hospital Universitario Nuestra Señora de Candelaria (Santa Cruz de Tenerife, Spain). Their main features are shown in Table 1. The study was approved by the local Ethics Committee (C.P. MO—C.I. PI-57/17 and C.P. MO—C.I. PI-39/14).

### 2.2. Candidate Genes

Four reference candidate genes were selected out of a set of significant genes previously reported in the literature [20,21,22,23,24,25,26,27,28]: *ACTB*, *TFRC*, *HPRT1* and *TBP* (Table 2).

### 2.3. RNA Isolation and Integrity

From each healthy skin and tumour paraffin block, 3–6 sections of 5–20 µm were sliced for histochemical detection. We discarded the first slice to avoid contamination. Subsequently, all sections were macrodissected before RNA purification. RNA was isolate from FFPE tissue using the “RNeasy FFPE kit” (Qiagen Inc., Hilden, Germany). We used a deparaffinizer solution recommended by the supplier to increase the amount of RNA and its integrity. RNA concentration was measured using the NanoDrop ND-1000 spectrophotometer (Thermo Fisher Scientific Inc., Waltham, MA, USA). The integrity of the RNA was confirmed by the amplification of the human *ACTB* gene by conventional PCR once the RNA was transcribed. Therefore, primers pair that amplified a fragment as short as of 60 bp were designed using the Bioinformatics tool Primers3 v. 0.4.0 software. Primers extended through two exons of the human *ACTB* gene to rule out contamination by genomic DNA. The primers sequences were as follows: FW 5′-CTCTTCCAGCCTTCCTTCCT-3′ and RV 5′-TTGAAGGTAGTTTCGTGGATG-3′.

### 2.4. Retrotranscription and Preamplification

For the synthesis of the second strand of RNA we used the “High Capacity cDNA Reverse Transcription Kit” (Thermo Fisher Scientific Inc., Waltham, MA, USA). The obtained cDNA was preamplified using a TaqMan^®^ PreAmp Master Mix (Thermo Fisher Scientific Inc., Waltham, MA, USA), which amplifies small amounts of cDNA without introducing amplification bias. The concentration used to carry out the pre-amplifications was 8 ng/µL, diluted 1:5 and run in a thermocycler under the following conditions: 14 cycles of 95 °C for 15 s and 60 °C for 4 min (after activation of the polymerase at 95 °C for 10 min). Primer pairs and specific probes of interest were used at a concentration of 0.05× in a final 10 µL pre-amp reaction.

### 2.5. Reference Genes Expression by qPCR

The relative gene expression quantification was performed using a “TaqMan Gene Expression Master Mix” (10×) (Applied Biosystems, Foster City, CA, USA) in a final reaction volume of 10 µL in a Step One Plus (Applied Biosystems, Foster City, CA, USA) real-time PCR detection machine. TaqMan predesigned probes (Thermo Fisher Scientific Inc., Waltham, MA, USA) for the four housekeeping *ACTB*/*TFRC*/*HPRT1*/*TBP* genes that were used for this study (Table 2). Every sample was run in triplicate. A non-template control was included in every reaction. A control sample was used as an internal calibrator and run in every plate to normalize for inter-plate variation.

### 2.6. Statistical Analysis

Clinical features of the samples are shown as: relative frequency of each category, 50th percentiles (5–95th), and non-normal and average scale ± SD, as appropriate. For the comparative analysis, a *t*-Student, chi-squared, Fisher exact, and a Kruskal–Wallis test were used to analyse differences in means and proportions of gene expression between studied groups. The statistical analysis was carried out with the SPSS vs. 25.0 software (IBM Corp, Armonk, NY, USA).

Bestkeeper software (Technical University of Munich, Germany) [29], NormFinder algorithm (Aarhus University Hospital, Denmark) [30] and the direct comparison method between means and standard deviations of the relative gene expression between different samples tissues were used. *p*-values < 0.05 were considered as statistically significant.

## 3. Results

### 3.1. Primer Specificity and RT-qPCR Amplification Efficiency

**Figure 1:**
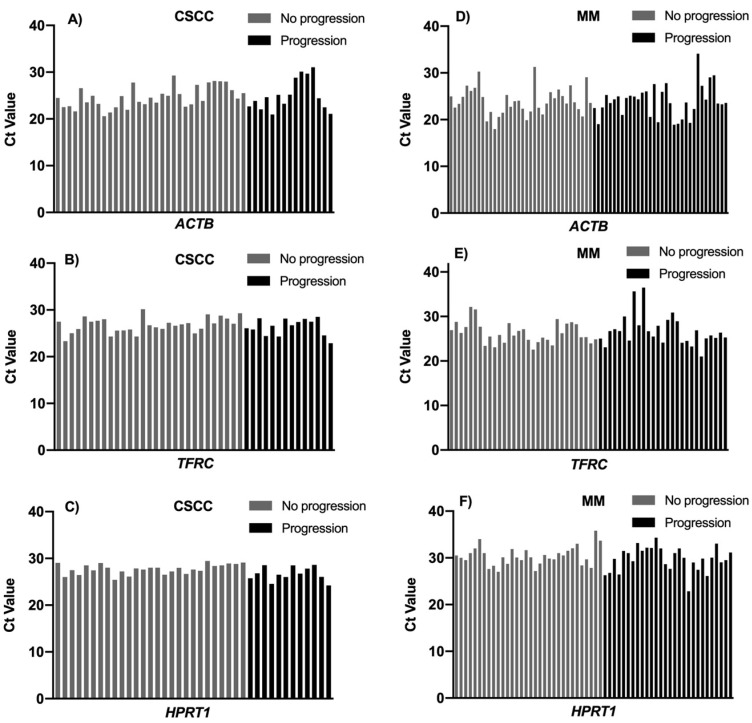
(**A**–**F**): Distribution of the raw Ct values of *ACTB*, *TFRC* and *HPRT1* in each of the analysed samples of CSCC and MM with and without progression.

*ACTB* gene expression was found in 100% of CSCC samples and 94.6% of MM samples. *TFRC* gene expression was detected in 87.7% of CSCC samples and in 80% of MM samples. *HPRT1* gene expression was detected in 80% of CSCC samples and 81% of MM samples (Figure 1). *TBP* was excluded for further analysis because its gene expression was detected in less than 40% of CSCC and MM samples.

### 3.2. Gene Expression Profiling 

**Figure 2:**
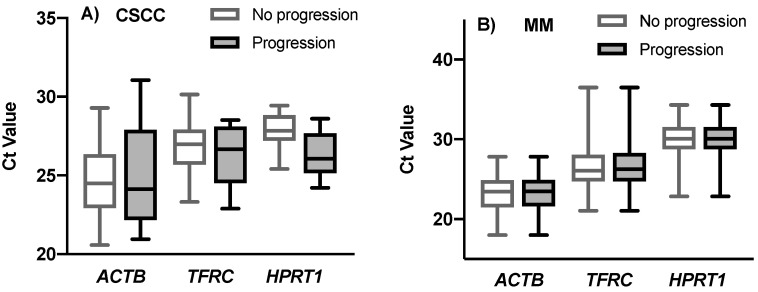
(**A**,**B**) Relative expression of the candidate reference genes *ACTB*, *TFRC* and *HPRT1* in MM and CSCC samples with progression vs. no progression of the disease. The boxes indicate median (25–75% percentiles) and the whiskers represent the minimum to maximum range.

The expression profiling of *ACTB*, *TFRC* and *HPRT1* as candidates reference genes in all samples was determine by the Ct value (cycle threshold) from RT-qPCR experiments. These three genes exhibited different expression levels across all samples. When comparing these genes, the Ct values ranged between 20.57 and 31.05 in CSCC samples (Figure 2). The minimum and maximum Ct values observed for MM tissues were 17.99 and 36.47 cycles, respectively.

### 3.3. Expression Stability of the Candidate Reference Gene

**Figure 3:**
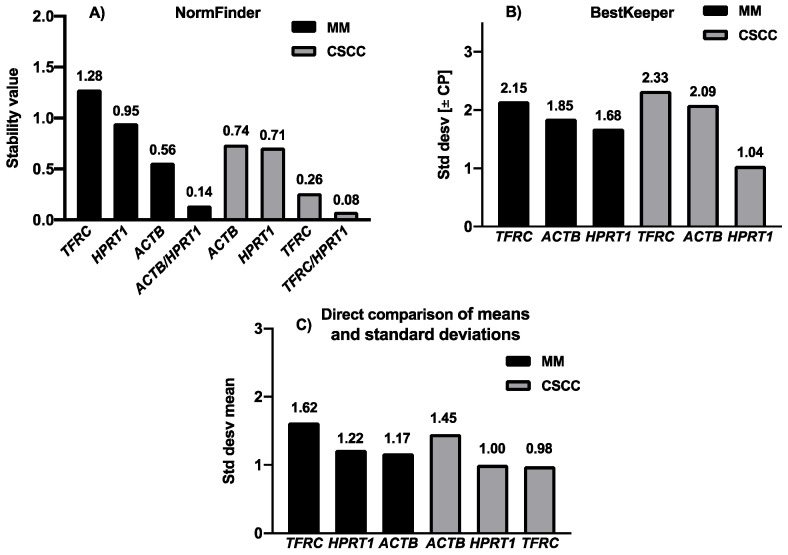
Candidate reference genes stability values (**A**). NormFinder analysis; lower values indicate high stability (**B**). BestKeeper analysis; lower SD of Ct values indicate less variable expression (**C**). Direct comparison of means and standard deviations: lower SD of Ct values indicate less variable expression.

The analysis with NormFinder software (Aarhus University Hospital, Denmark) showed that *ACTB* was the most stable gene in MM, and *TFRC* in CSCC samples, showing the lowest intra-group and inter-group expression variability in each case. For MM samples, the stability value was 0.56, and for CSCC it was 0.26 (Figure 3A). The stability analysis with BestKeeper software (Technical University of Munich, Germany) indicated that *HPRT1* was the most stably expressed gene in cutaneous MM tissues when compared to *ACTB* (1.68 vs. 1.85) and *TFRC* (1.68 vs. 2.15). The same was found for CSCC tissues, where *HPRT1* was the most stable expressed gene followed by *ACTB* (1.04 vs. 2.09) and *TFRC* (1.04 vs. 2.33) (Figure 3B). Using NormFinder software, the best combination of two reference genes for normalizing CSCC tissue were *TFRC* and *HPRT1* (stability value 0.08), and for MM, *HPRT1* and *ACTB* were the most stable combination (0.14) (Figure 3A). In these cases, while using the calculations required in the normalization method chosen (for example: 2^−^^△△Ct^ method), an average of the Ct values of the two reference genes must be calculated for each sample. Then, the resulting Ct value will be used for correcting the target gene Ct value.

When using direct comparison of means and standard deviations, we identified the intra and intergroup variability between CSCC and MM, with progression and without disease progression. The most stable expressed gene in MM cancer tissues was *ACTB* (Figure 1D and Figure 3C), with a lower average between mean SD and mean Ct (1.17 for *ACTB*; 1.22 for *HPRT1*; 1.62 for *TFRC*), while in CSCC cancer tissues it was *TFRC* (1.00 for *TFRC*; 1.13 for *HPRT1*; 1.45 for *ACTB*) (Figure 1C and Figure 3C).

## 4. Discussion

Our goal was to establish the best reference gene for studies involving gene expression analysis in CSCC and MM FFPE samples out of a pre-selected set of genes. We studied the potential role of *ACTB*, *TFRC*, *HPRT1* and *TBP* as reference genes. These genes have cell maintenance functions and are expressed in all cell types of an organism under physiological and pathological conditions. These constitutive genes are essential for the preservation of basic cellular functions [31].

We demonstrated that *HPRT1* was the most stable reference gene in CSCC and MM FFPE tissue in comparison with *ACTB* or *TFRC* when we used Bestkeeper software. On the other hand, when using NormFinder software or direct comparison of means and standard deviations, *ACTB* and *TFRC* were the most appropriate genes for analysing gene expression in FFPE samples of MM and CSCC, respectively. These three genes were widely used in previous expression studies [7,22,24,32] in MM skin samples, but not in CSCC tissues.

Recently, Christensen et al., tested 24 possible reference genes for gene expression studies in MM FFPE samples and purposed a combined geometric mean of *CLTA*, *MRPL19* and *ACTB* expression levels as the most adequate formula for normalization of gene expression studies in MM tissue [7]. They reported that *ACTB* alone is not adequate as a gene expression normalizer. Our results agree with this study because our analyses significantly improve the variation when we used the combination of *ACTB* and *HPRT1*, so this result supports their use in further gene expression studies in MM tumour samples for more reliable results.

*TFRC* has been studied as a reference gene in several types of tissues and different diseases showing to be stably expressed in breast, lung and pancreatic cancers [21,33]. However, little is known about its expression in skin cancer. To our knowledge, there is solely a study that investigated its expression in FFPE and frozen MM samples of 25 patients with primary melanoma and cutaneous or lymph node MM metastases. *TFRC* was the one that showed a minimum mean coefficient of variation among other genes [22]. In contrast to this, our results found *TFRC* to be the least stable gene beyond *ACTB* and *HPRT1* for MM, but the most stable one in CSCC. Moreover, we found that the best combination of two reference genes for normalizing CSCC tissue was *TFRC* and *HPRT1*. To our knowledge, this is the first study that investigated the role of *TFRC* and *HPRT1* as potential useful reference genes for expression studies in CSCC tumour FFPE samples.

The NormFinder algorithm only analyses samples with significant signal detection. Thus, samples without intense *HPRT1* and *TFRC* signal were lost. BestKeeper software has the advantage of including into the analysis lost Ct values due to low signal, and therefore a larger number of samples can be analysed. The method of direct comparison of Ct and standard deviation between tissues allows overview of gene stability. It is a very simple but useful technique to detect the stability of the reference genes. Combined use of these methods together with the BestKeeper algorithm allowed us to determine which of these genes is the most stable, and which is the best combination between them for CSCC and MM FFPE sample gene expression studies. Therefore, it is highly recommended to use at least two or three methods to validate appropriate reference genes for expression studies specific for different samples, as in this study.

Fixation and paraffin embedding of the tissue influence both the yield and quality of RNA [22,34,35], especially when archival FFPE tissue is used, where storage and conservation conditions are unknown. There is no method that is easily capable of accurately assessing the quality of these types of samples. We suggest that the best method to assess a sample´s RNA quality is to simply determine if it generates expression data [36,37]. A main issue when dealing with RNA isolation from FFPE tissues is the pre-amplification step [38]. One of the strengths of the current study was to use a pre-amplification cDNA procedure that allows amplification of cDNA with no bias, and provides extremely high correlation between amplified and unamplified cDNA [38].

The current study has several limitations. On the one hand, *TBP* were excluded from analysis because of the low percentage of detection in CSCC and MM samples. This may be due to chemical modifications during fixation and storage of the tissues. However, gene deregulation along tumour progression may also account for the finding [39]. On the other hand, mRNA quality was measured in a spectrophotometer and confirmed by conventional PCR of the *ACTB* gene from the cDNA. It allowed us to preliminary evaluate the mRNA and to discard poor quality samples. Lack of RNA validation entails the possibility that gene expression data may be equivocal because of the routine fixation of the sample or the RNA purification method.

## 5. Conclusions

Skin cancer research focuses on genes involved in disease progression. Gene expression studies allow a rapid analysis for candidate tumour markers that can accurately detect tumour progression [40,41]. Following the MIQE guidelines for the publication of RT-qPCR experiments [42], it is crucial to assess reference genes properly. We suggest that *ACTB* and *HPRT1* in combination are the appropriate reference genes for the normalization of gene expression studies in MM FFPE samples, as is the combination of *TFRC* and *HPRT1* reference genes in CSCC FFPE tissue.

## Figures and Tables

**Table 1:** Main clinic-pathological features of included individuals.

		Melanoma *	Squamous Cell Carcinoma ^+^
**Number of Patients**		74	49
**Male/Female Ratio**		0.8	1.6
**Median Age at Diagnosis (y)**		68	75
**Location *n* (%)**	Trunk	13 (18)	3 (6)
	Limbs	47 (63)	8 (16)
	Head or neck	14 (19)	38 (78)
**Stage ^#^ *n* (%)**	I–II	53 (72)	49 (100)
	III–IV	21 (28)	0 (0)
**Disease Progression *n* (%)**		36 (49)	13 (27)

^#^ Stage at diagnosis. * Cutaneous primary malignant melanoma. ^+^ Cutaneous primary squamous cell carcinoma.

**Table 2:** Candidates for reference genes in CSCC and MM FFPE samples.

Gene	Tittle	Accession No.	Amplicon Size (bp ^#^)	TaqMan Assay
*ACTB*	Beta-actin	NM_001101	63	Hs01060665_g1
*TFRC*	Transferrin receptor	NM_003234	66	Hs00951083_m1
*HPRT1*	Hypoxanthine phosphoribosyltransferase 1	NM_000194	82	Hs02800695_m1
*TBP*	TATA-box binding protein	NM_001172085	91	Hs00427620_m1

^#^ bp: base pair.

## Data Availability

All data relevant to the study are included in the article. Ct raw data.
